# Targeting CD13 (aminopeptidase-N) in turn downregulates ADAM17 by internalization in acute myeloid leukaemia cells

**DOI:** 10.18632/oncotarget.1788

**Published:** 2014-02-18

**Authors:** Sandrine Bouchet, Ruoping Tang, Fanny Fava, Ollivier Legrand, Brigitte Bauvois

**Affiliations:** ^1^ INSERM U1138, Université Pierre et Marie Curie, Université Paris-Descartes, Centre de Recherche des Cordeliers, Paris, France; ^2^ INSERM U938, Centre de Recherche de Saint-Antoine, Paris, France; ^3^ Service d'Hématologie, Hôpital St Antoine, Paris, France

**Keywords:** myeloid leukaemia, a disintegrin and metalloproteinase, matrix metalloproteinase, tumour necrosis factor-α-converting enzyme, oncogenesis

## Abstract

Secreted matrix metalloproteinases (MMP)-2 and MMP-9 and membrane-anchored aminopeptidase-N/CD13 are abnormally expressed in human acute myeloid leukaemia (AML). We previously showed that CD13 ligation by anti-CD13 monoclonal antibodies can induce apoptosis in AML cells. Here, we assessed ADAM17 expression in primary blood blasts CD13^+^CD33^+^ from patients with AML. Primary AML cells expressed ADAM17 transcript and its surface expression was higher in subtype M4 (myelomonocytic) and M5 (monocytic) AML specimens than in M0 and M1/M2 (early and granulocytic) specimens. In AML cell lines defining distinct AML subfamilies (HL-60/M2, NB4/M3, THP-1/M5, U937/M5) and primary AML cells cultured ex vivo, anti-CD13 antibodies downregulated surface CD13 and ADAM17 without affecting MMP-2/-9 release. Knockdown of CD13 by siRNA prevented anti-CD13-mediated ADAM17 downregulation, indicating that CD13 is required for ADAM17 downregulation. Soluble ADAM17 was not detected in the medium of anti-CD13 treated cells, suggesting that ADAM17 was not shed. After ligation by anti-CD13, CD13 and ADAM17 were internalized. Subsequently, we found that ADAM17 interacts with CD13. We postulate that the interaction of ADAM17 with CD13 and its downregulation following CD13 engagement has important implications in AML for the known roles of ADAM17 in tumour-associated cell growth, migration and invasion.

## INTRODUCTION

Acute myeloid leukaemia (AML) is a clinically and genetically heterogeneous haematopoietic cancer characterized by the clonal accumulation of immature myeloid precursors in the bone marrow [1, 2]. Distinct AML subfamilies are defined by the development stage at which the cells are arrested [1, 2]. Human AML cells show abnormally high levels of proliferation and survival and infiltrate extramedullary organs. The majority of AML respond to initial treatment; however relapse is common indicating resistance of malignant cells to chemotherapy [1, 2]. There is now compelling evidence that deregulated interactions between malignant cells, surrounding stromal cells and the extracellular matrix (ECM) play a pivotal role in survival and drug resistance of tumour cells [3, 4]. Several types of proteases control the degradation and turnover of ECM components [5-8]. The secreted matrix metalloproteinases (MMPs) (including MMP-2 and MP-9) and membrane-anchored ADAMs (a disintegrin and metalloproteinase) (including ADAM17, also known as tumour necrosis factor-α-converting enzyme) cleave many different targets (ECM, cytokines, growth factors, chemokines and cytokine/growth factor receptors) [9-12]. Through their proteolytic activities, these proteases are implicated in tumour-associated processes such as cell growth, survival, migration, invasion and angiogenesis [11, 12]. There is now compelling evidence to suggest that by binding cell surface proteins, secreted MMP-2 and MMP-9 can directly trigger intracellular signalling pathways that control tumour cell events [13]. Another family of membrane-anchored enzymes (the ectopeptidases, including the metalloenzyme aminopeptidase-N/CD13) have been shown to participate in extracellular proteolysis and influence major biological processes (cell growth, motility and the secretion of inflammatory and angiogenic cytokines) [5, 14, 15].

The metalloproteases MMP-2, MMP-9, CD13 and ADAM17 are already considered to be useful markers in several cancers [10, 16-19]. In contrast to normal myeloid precursors, AML cells secrete high levels of the latent forms of MMP-2 and/or MMP-9 (proMMP-2, proMMP-9) [20-24]. Both proMMP-2 and proMMP-9 may contribute to the dissemination of AML cells from the bone marrow [21, 25]. In AML, bone marrow levels of MMP-9 are lower in patients who achieve a complete remission than in patients who do not [26]. The CD13 antigen is strongly expressed on stem cells and leukaemic blasts in all AML subtypes [27]. We recently showed that monoclonal antibodies against CD13 can induce caspase-dependent apoptosis in AML cells (independently of CD13 enzymatic activity) [28]; these results highlighted CD13 as a potential drug target in AML [18]. ADAM17 is expressed in human AML cell lines derived from AML [29, 30]. However, to the best of our knowledge, there are no literature data on ADAM17 expression in primary AML cells. In the present study, we first investigated the expression status of ADAM17 in myeloid blasts from peripheral blood as a function of the latter's French-American-British (FAB) subtype (M0, M1, M2, M4, M5). In addition, we sought to determine whether CD13 ligation could affect the *ex vivo* expression of both proMMP-2/-9 and ADAM17 by primary cells from patients with AML. We demonstrate herein that ADAM17 is expressed in primary AML cells, identified a novel CD13-ADAM17 interaction and then provided evidence that CD13 ligation downregulates ADAM17 surface expression in AML.

## RESULTS

### Expression of ADAM17, CD13, MMP-2 and MMP-9 in primary AML cells

**Figure 1:**
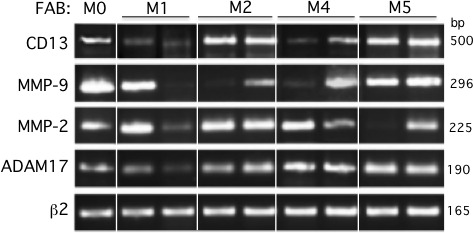
PCR analyses of CD13, MMP-9, MMP-2 and ADAM17 transcripts in primary AML cells Samples were standardized for total cDNA content by assessing the presence of identical amounts of β2-microglobulin transcripts. PCR products were run on 1.8% agarose gels.

**Figure 2:**
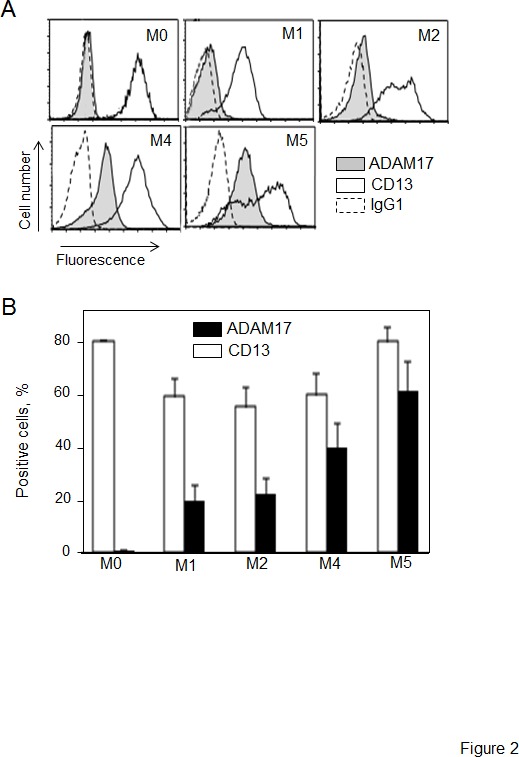
Levels of surface CD13 and ADAM17 expression in primary AML cells (A) Representative histograms of M0-, M1-, M2-, M4- and M5-subtype primary AML cells stained with anti-CD13-PE and anti-ADAM17-PE and then examined by flow cytometry analysis. Staining of cells with their isotype IgG1-PE served as the negative control (broken line). (B) Results of the percentage of surface CD13 and ADAM17 expression on AML blast samples (1 M0, 18 M1, 12 M2, 12 M4, 9 M5). Values are expressed as means ± SEM.

**Figure 3:**
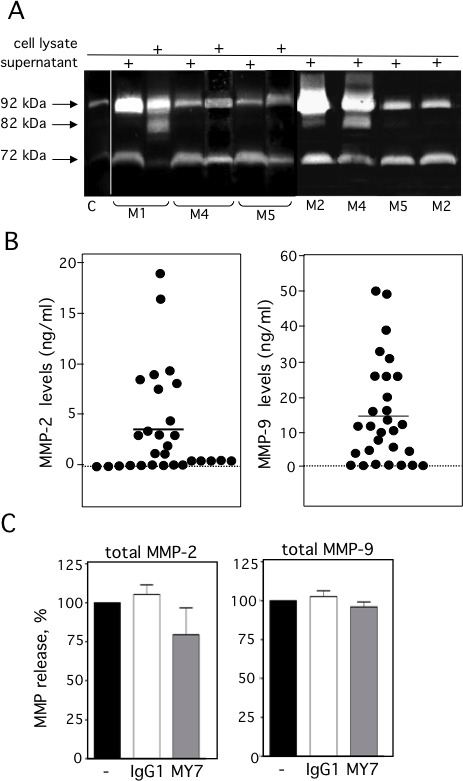
Expression of proMMP-2 and proMMP-9 in AML cells (A) The gelatinolytic activities of MMP-2 and MMP-9 were analyzed using zymography, in the 48 h-conditioned media (supernatant) and/or in whole cell lysates from 7 patients with AML. Control (C) FCS-supplemented culture medium alone incubated under the same conditions. (B) Total MMP-2 (first column) and total MMP-9 (second column) productions in the 48 h-culture supernatants from 29 AML samples were determined by ELISA. Mean concentrations are indicated by a horizontal line. Control included FCS-supplemented culture medium alone incubated under the same conditions. (C) AML cells were cultured for 48 h in the presence of absence of IgG1 or MY7 (10 μg/ml). Total MMP-2 and MMP-9 production measured by ELISA. Data represent the mean of six AML samples. Values are expressed as means ± SEM.

We examined the levels of ADAM17, CD13, MMP-2 and MMP-9 on primary AML blood blasts with different subtypes (M0, M1, M2, M4, M5). Representative examples of RT-PCR products are shown in Figure 1. CD13 and ADAM17 PCR products were detected in all the AML samples tested (Figure 1). In contrast, the MMP-2 and MMP-9 transcripts patterns appeared to be independent of the FAB subtype (Figure 1). Figure 2A shows the representative results of flow cytometry for M0-, M1-, M2-, M4- and M5-subtype primary AML cells. As previously reported [27], all AML samples express surface high levels of CD13 (Figure 2A). However, surface levels of ADAM17 were lower for FAB M0, M1, M2 AML cells than for FAB M4/M5 cells (Figure 2A). There were statistically significant ADAM17 differences in the number of fluorescent cells (Figure 2B) and the mean of fluorescence intensity (data not shown) of the blasts from 52 patients with various FAB subtypes of AML. Thus, the ADAM17 mRNA levels in AML blasts appeared to be correlated with the levels of surface ADAM17 protein. In parallel, zymography analysis of AML cell lysates and their conditioned culture media (after 48 h of culture) revealed the presence of proMMP-9 and proMMP-2 activities at 92 kDa and 72 kDa respectively (Figure 3A). Active MMP-9 (at 82 kDa) was detected in some samples (Figure 3A). As quantified in ELISAs, the mean (range) MMP-2 and MMP-9 concentrations (after a 48 h of culture) released by AML cells were respectively 3,4 (0-18) and 14,4 (0-51) ng/ml (Figure 3B).

### CD13 ligation induces ADAM17 downregulation in primary AML cells

**Figure 4:**
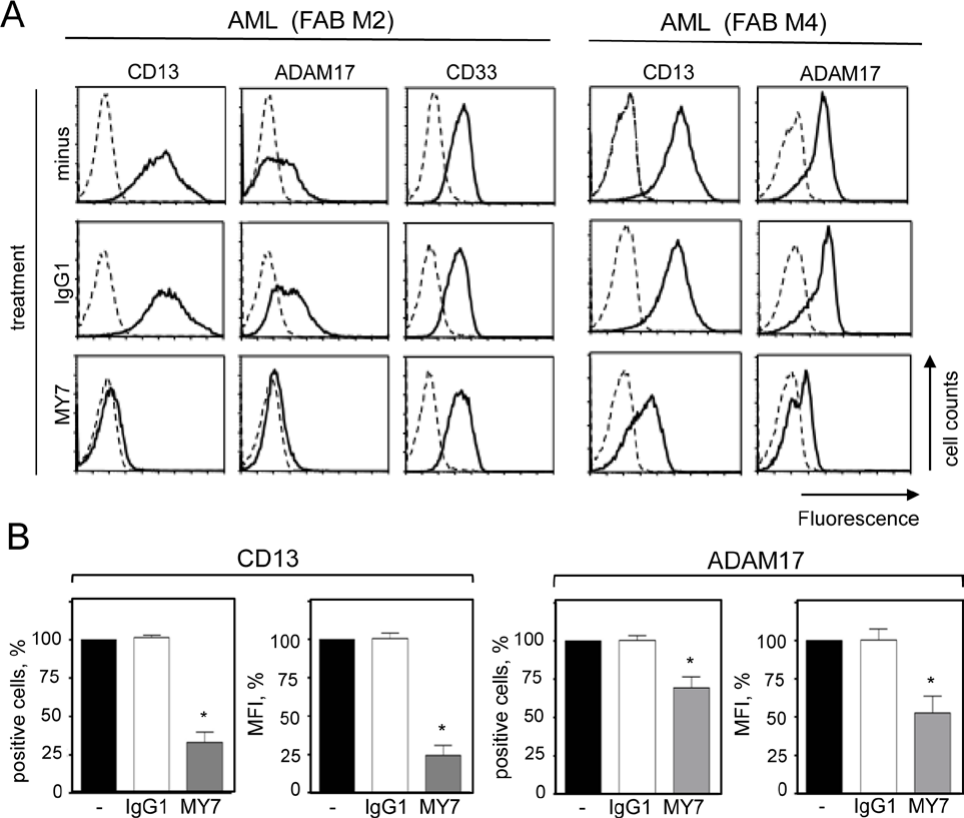
Effect of the anti-CD13 MY7 on surface CD13 and ADAM17 expression in primary AML cells Cells were cultured for 24 h in the presence of absence of mIgG1 or MY7 (10 μg/ml). (A) Cells were stained with anti-CD13-PE, anti-ADAM17-PE, anti-CD33-FITC or their isotypes (-PE, -FITC) and then examined by flow cytometry. (B) Results of the percentage of CD13- and ADAM17-positive AML cells and of MFI. Values are expressed as means ± SEM (n = 15, all FAB subtypes considered). *P <0,0001 vs IgG1-treated cells.

The specific monoclonal antibodies (mAbs) WM15, SJ1D1 and MY7 which recognize different epitopes of CD13 [31-33] bind similar levels of surface CD13 on primary AML cells [28]. We further examined the effects of MY7 anti-CD13 on the levels of released proMMP-2/-9 and surface CD13 and ADAM17 in AML blasts. Cells were cultured in the absence or presence of MY7 or its isotype-matched IgG1 (10 μg/ml) (effective concentration for inducing AML cell apoptosis [28]). As assessed by ELISAs, the amounts of proMMP-2 and proMMP-9 released by AML cells were not significantly affected by 48 h of MY7 treatment (Figure 3C) or WM15 and SJ1D1 treatment. As examplified in Figure 4A, 24 h of exposure to MY7 induced the concomitant downregulation of CD13 and ADAM17 in AML samples. These results were confirmed in all primary AML cells and did not appear to depend on the FAB subtype (Figure 4B). Other antigens tested (such as CD15, CD33, CD44, CD64, CD143/angiotensin converting enzyme and integrins β1/β2) were not affected by MY7 treatment (Figure 4A for CD33, and data not shown). The MY7-responsive samples also responded to WM15 or SJ1D1 by downregulating surface CD13 and ADAM17.

### CD13 ligation induces ADAM17 downregulation in AML cell lines

**Figure 5:**
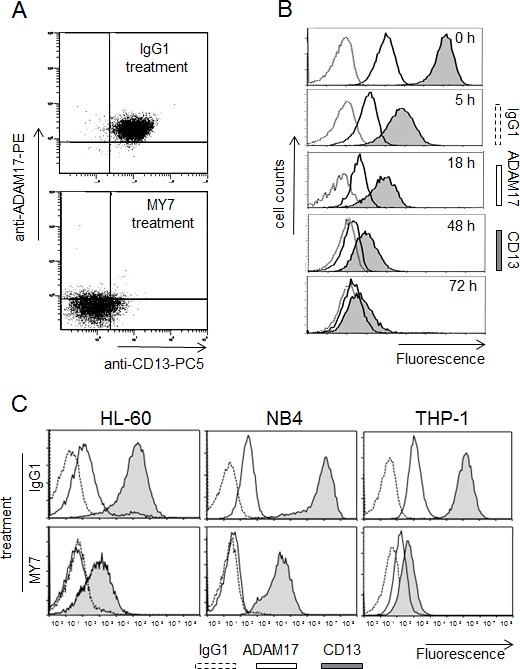
MY7-mediated downregulation of CD13 and ADAM17 in AML cell lines (A) U937 cells were cultured for 48 h in the presence of absence of mIgG1 or MY7 (10 μg/ml). Cells were stained with mIgG1-PC5/mIgG1-PE or anti-CD13-PC5/anti-ADAM17-PE and then examined by flow cytometry. Quadrants delineated by squares indicate negative and positive populations of cells as determined using negative control (mIgG1-PC5/mIgG1-PE). (B) Cells were cultured in the presence of absence of mIgG1 or MY7 (10 μg/ml) for 5, 18, 48 and 72 h. Time-response histograms showing specific fluorescence intensities of CD13 and ADAM17 of MY7-treated cells as determined by flow cytometry. (C) AML cell lines HL-60, NB4 and THP-1 were treated with mIgG1 or MY7 (10 μg/ml) for 18 h. Cells were stained with anti-CD13 MY7-PE, anti-ADAM17-PE or mIgG1-PE (negative control) and analyzed for surface CD13 and ADAM17 expression. Representative experiments are shown.

We first examined the effects of anti-CD13 on ADAM17 expression in monoblastic (M5) U937 cells. Untreated U937 cells co-expressed CD13 and ADAM17. Surface CD13 and ADAM17 levels both fell after 48 h of incubation with MY7 but did not change in IgG1-treated cells (10 μg/ml) (Figure 5A) or untreated cells (data not shown). Time-course studies revealed a time-dependent inhibitory effect of MY7 on the surface CD13 and ADAM17 levels; a significant effect was already noted in MY7-treated cells after 5 h of incubation and persisted at 72 h (Figure 5B), indicating that the downregulation of CD13 and ADAM17 proteins by MY7 is both rapid and long-lasting. Moreover, the WM15 and SJ1D1 mAbs were just as efficient as MY7 in downregulating surface ADAM17 expression. The other AML cell lines HL-60 (myeloblastic/M2), NB4 (promyelocytic/M3) and THP-1 (monoblastic/M5) coexpressed surface CD13 and ADAM17, whose levels fell on MY7-treated cells but did not change on untreated or IgG1-treated cells (Figure 5C). We therefore decided to investigate the molecular mechanisms underlying the downregulation of ADAM17 by anti-CD13 treatment in U937 cells.

### MY7-mediated CD13 and ADAM17 internalization in U937 cells - CD13/ADAM17 association

**Figure 6:**
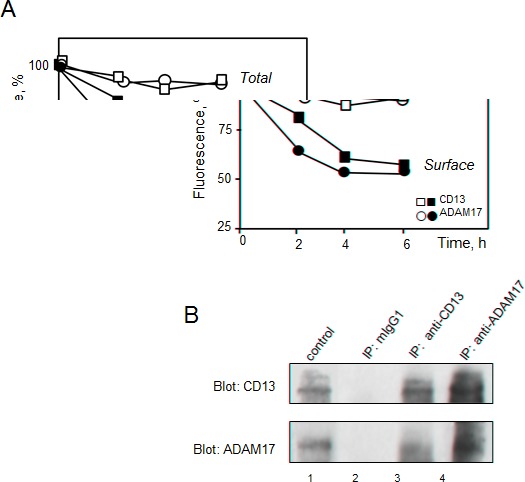
MY7-mediated internalization of CD13 and ADAM17; CD13/ADAM17 association (A) U937 cells were pulsed with anti-CD13 MY7-PE, anti-ADAM17-PE or mIgG1-PE (negative control) at 4°C, then cultured for 2, 4 and 6 h before analysis for total fluorescence. In separate samples, cells were cultured with MY7 (or its isotype IgG1) for the same times periods, then stained with anti-CD13 MY7-PE, anti-ADAM17-PE or mIgG1-PE (negative control) and analyzed for surface CD13 and ADAM17 expression. Results are expressed as the percentage of the mean fluorescence intensity in MY-7 treated cells vs IgG1-treated cells. (B) U937 cells were lysed, and the lysates were immunoprecipitated with either mIgG1 (lane 2), anti-CD13 (MY7) (lane 3) or anti-ADAM17 (R&D1362) (lane 4). Immunoprecipitates (IP) were washed and separated by SDS/PAGE under reducing conditions and immunoblotted with anti-CD13 (SJ1D1) or anti-ADAM17 (Cell Signalling 3976) to evaluate CD13/ADAM17 interaction. The whole cell lysate served as control.

Soluble forms of ADAM17 are found in biological fluids [5]. We used ELISAs to establish whether down-regulation of ADAM17 by anti-CD13 treatment was accompanied by release of soluble ADAM17 into the medium by MY7-treated cells (AML cell lines and primary cells). However, no soluble ADAM17 was detected (data not shown). Next, U937 cells were pulsed with anti-CD13 MY7-PE or anti-ADAM17-PE at 4°C, then cultured for short periods of time (2, 4, 6 h) before to be analyzed for total CD13 and ADAM17 expression. In separate samples, cells were cultured with MY7 (or its isotype IgG1) for the same time periods, then stained with anti-CD13 MY7-PE or anti-ADAM17-PE and analyzed for surface CD13 and ADAM17 expression. A flow cytometry analysis showed that the amount of total CD13 and ADAM17 did not change significantly over time, whereas the levels of surface CD13 and ADAM17 fell significantly (Figure 6A). These data strongly suggest that CD13 ligation led to the internalization of CD13 and ADAM17. In an attempt to characterize the mechanism of ADAM17 and CD13 endocytosis in MY7-treated cells, we measured the effects of pitstop 2 (a potent inhibitor of both clathrin-dependent and independent-endocytosis [34]) on MY7-mediated ADAM17 downregulation in U937 cells. Surprisingly, 10 μM pitstop 2 was associated with accentuated MY7-mediated ADAM17 downregulation, rather than inhibition (data not shown). We further investigated the relationship between CD13 and ADAM17. Co-immunoprecipitation experiments on U937 cells were performed in order to screen for CD13/ADAM17 interactions. Whole cell lysates were subjected to co-immunoprecipitation with anti-CD13 MY7, anti-ADAM17 or the corresponding mIgG1 isotype. A 135 kDa protein (corresponding to the molecular mass of ADAM17) was co-immunoprecipitated with CD13 (Figure 6B lane 3). Conversely, a 105 kDa protein (corresponding to the molecular mass of CD13) co-immunoprecipitated with ADAM17 (Figure 6B lane 4). No protein was detected when immunoprecipitation was carried with the isotype (Figure 6B lane 2). Taken as a whole, these data indicate that the MY7-mediated downregulation of surface CD13 and ADAM17 resulted in the intracellular accumulation of these proteins. The immunoprecipitation data suggest that ADAM17 interacts directly with CD13.

### A lack of CD13 prevents MY7-mediated ADAM17 downregulation

**Figure 7:**
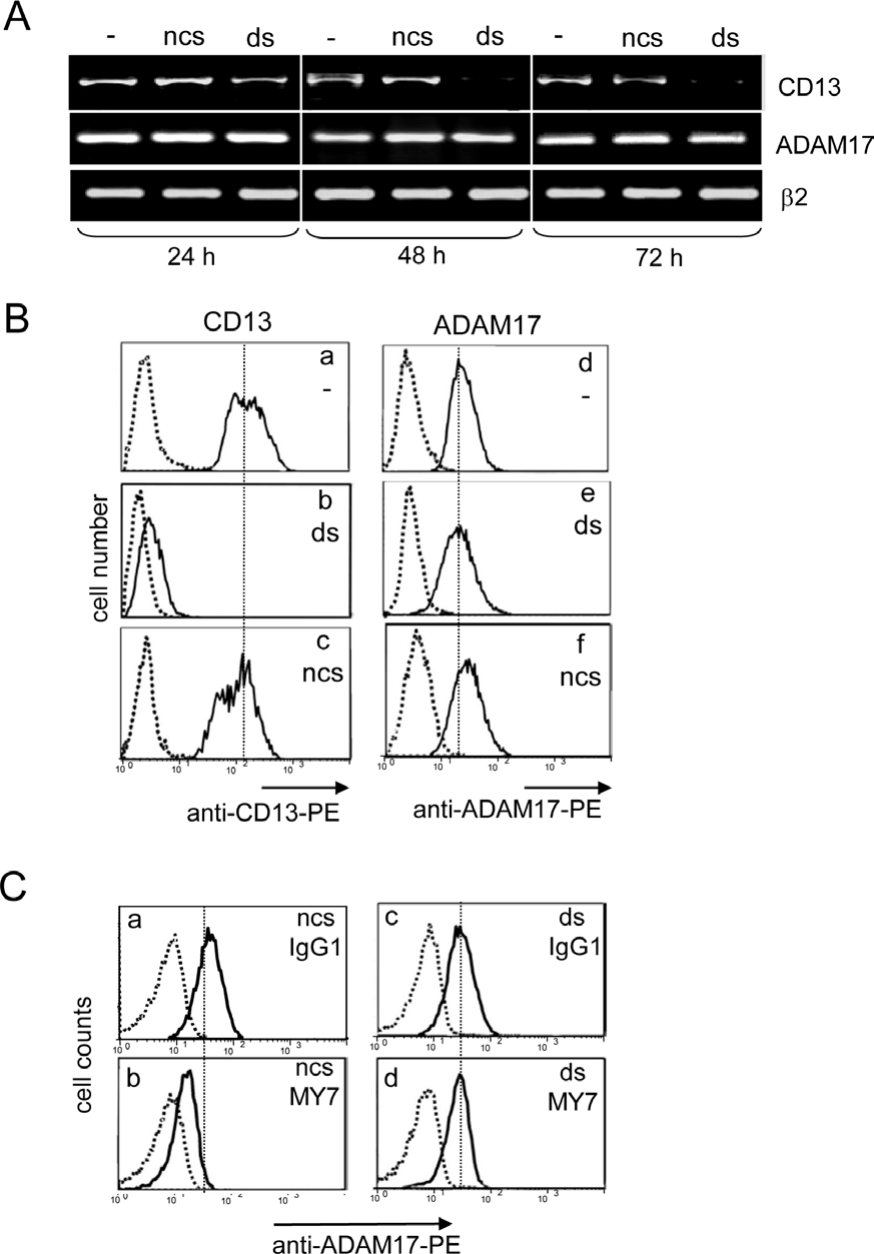
Impact of CD13 inhibition by siRNA on MY7-mediated ADAM17 downregulation in U937 cells (A) U937 cells were cultured for 24, 48 or 72 h, before or after transfection with 100 nM CD13 specific siRNA (ds-cells) or negative control siRNA (ncs-cells). Then, the cDNAs were used as templates for PCR reactions using specific primers for CD13, ADAM-17 or β2-microglobulin. (B) Surface CD13 and ADAM17 levels in ncs-cells and ds-cells after 48 h siRNA transfection were measured by cytometry with anti-CD13-PE or anti-ADAM17-PE or their isotype mIgG1-PE. (C) After 48 h transfection, ds-cells and ncs-cells were cultured for 24 h in the presence of absence of mIgG1 or MY7 (10 μg/ml). Surface ADAM17 levels were measured by cytometry with anti-ADAM17-PE or its isotype mIgG1-PE.

Anti-CD13 small interfering RNA (siRNA) experiments were carried out in order to confirm CD13's contribution to the MY7-mediated downregulation of ADAM17. Hence, CD13 duplex siRNA (ds) and negative control siRNA (ncs) were tested for their ability to specifically suppress CD13 in U937 cells. Preliminary experiments showed that 100 nM ds was the most effective concentration for inhibiting surface expression of CD13 (data not shown). In an RT-PCR analysis, time-dependent downregulation of CD13 transcription was observed in ds-treated U937 cells (Figure 7A). In contrast, ncs had no effect on CD13 transcript expression levels, which were the same as in untreated cells (Figure 7A). Flow cytometry analysis indicated that the levels of surface CD13 were significantly lower after 48 hours post-transfection in ds-treated cells (Figure 7B panel b) than in untreated cells and ncs-treated cells (Figure 7B panels a and c). Downregulation of surface CD13 expression was associated with loss of CD13 enzymatic activity in ds-U937 cells, relative to untreated cells and ncs-treated cells (data not shown). In contrast, levels of ADAM17 mRNA and protein were not affected, which demonstrated the specificity of the anti-CD13 siRNA (Figure 7A & B panels d, e and f). After 48 h of treatment with ds or ncs, cells were incubated with MY7 (10 μg/ml) for 24 hours at 37°C. As seen in Figure 7C, incubation with MY7 decreased surface ADAM17 levels on ncs-treated cells (panel b compared to panel a) but not on ds-treated cells (panel d compared to panel c); this finding indicates that the MY7-mediated downregulation of ADAM17 was disrupted by CD13 silencing. In addition, untreated U937 cells released low levels of proMMP-9, which were not affected in ncs- and ds-treated cells by up to 72 hours of MY7 treatment (data not shown). Taken as a whole, these data indicate that CD13 is required for MY7-mediated ADAM17 downregulation.

## DISCUSSION

Our present study has provided first evidence that AML cells synthesize ADAM17 and express it at their surface. Another major finding of our study is the striking downregulation of ADAM17 following CD13 ligation by anti-CD13 mAbs. Our results strongly suggest that CD13 binds to ADAM17 and thus provide evidence of a regulatory role for CD13 engagement in ADAM17 downregulation in AML cells.

AML blast cells from blood (and from bone marrow; data not shown) express detectable levels of ADAM17 mRNA and surface protein. Levels of ADAM17 appeared to depend on the differentiation stage, since they were higher in M4 and M5 cells (myelomonocytic and monocytic cells, respectively) than in the early and granulocytic M0/M1/M2 cells. In contrast, proMMP-2/-9 patterns were not subtype-restricted. Our data agree with studies in which proMMP-2/-9 levels and FAB subtypes were not correlated [20, 35]. It remains to be seen whether ADAM17 levels are predictive of chemoresistance or responsiveness to treatment in AML.

Like primary AML cells, the representative AML cell lines HL-60 (M2), NB4 (M3), THP-1 and U937 (M5) coexpressed surface CD13 and ADAM17. Surface CD13 targeting by specific anti-CD13 mAbs (MY7, SJ1D1 and WM15) induces AML cell death in primary and cell lines [28] (data not shown). We further examined the effects of these anti-CD13 mAbs on the levels of proMMP-2/-9 and ADAM17 expressed by AML cells. None of the three anti-CD13 (when used at a dose of 10 μg/ml *in vitro*) had a significant effect on the release of proMMP-2 and proMMP-9 by AML cells. In contrast, ADAM17 downregulation was induced by all three CD13 mAbs (again at 10 μg/ml) after as few as 2 h and persisted until 72 h. Non-stimulated or MY7-stimulated AML cells did not produce detectable amounts of soluble ADAM17, suggesting that ADAM17 downregulation was not a consequence of ADAM17 shedding. Hence, it is likely that MY7-induced downregulation of surface ADAM17 occurs *via* internalization. Our previous work showed that type II interferon (IFN)-γ downregulated and internalized surface ADAM17 in myeloid cells [29]. Myeloid cells are known to produce type I IFNs, whereas IFN-γ expression is restricted to lymphoid cells [36]. However, a recent study of bacterial pneumonia showed that human neutrophils are able to produce IFN-γ [37]; it is therefore possible that other myeloid cells can produce IFN-γ when stimulated appropriately. It remains to be seen whether MY7 might induce an autocrine loop with IFN-γ in U937 cells. However, soluble IFN-γ was not detected in the conditioned media of MY7-treated U937 and AML blasts or in plasma from AML patients (data not shown). This rules out the involvement of endogenous IFN-γ in MY7's inhibition of ADAM17 expression.

Furthermore, we showed that the siRNA silencing of CD13 consistently prevented the MY7-mediated downregulation of ADAM17 in U937 cells, thus indicating that CD13 is required for MY7-mediated ADAM17 downregulation. In general, the modulation of antigen levels by a cognate mAb leads to cell internalization of antigen-antibody complexes [38]. Given that anti-CD13 treatment induced the internalization of both CD13 and ADAM17, CD13/ADAM17 complexes may have formed. Accordingly, the results of our co-immunoprecipitation experiments strongly suggest that CD13 binds to ADAM17. We further explored the endocytic pathways by which CD13 and ADAM17 are internalized. Conventionally, endocytic mechanisms are primarily classified as being clathrin-dependent or -independent [39]. Recent research has revealed that clathrin-independent processes are quite diverse [39]. For example, the endocytosis of membrane proteins is caveola-dependent manner [39]. The cytoplasmic domain of human ADAM17 contains a potential motif (YESL) for clathrin-mediated endocytosis [40]. Studies of monocytes and epithelial and endothelial cells indicate that CD13 and ADAM17 are internalized via clathrin- and/or caveola-dependent pathways [41-44]. Surprisingly, pitstop 2 (inhibitor of both clathrin-dependent and independent-endocytosis) did not block MY7-mediated ADAM17 downregulation. This observation strongly suggests that CD13 and ADAM17 are not internalized *via* the classical clathrin-dependent pathway. It is not yet known whether pitstop 2 inhibits caveola-mediated endocytosis. Other mechanisms of clathrin-independent endocytosis have been observed for the internalization of glycolipid-binding toxins (such as shiga and cholera toxins), glycosylphosphatidylinositol (GPI)-anchored proteins, the epidermal growth factor receptor (under certain conditions) and a number of plasma membrane proteins [39]. Although pitstop 2 does not inhibit the endocytosis of shiga toxin [34], it is not yet known whether it can block the other above-mentioned internalization processes.

The reversion-inducing-cysteine-rich protein with Kazal motifs (RECK) is a unique, membrane-anchored glycoprotein that strongly inhibits a number of MMPs [45]. Both RECK and its target molecules are internalized via the GPI-anchored proteins enriched early endosomal compartments-mediated endocytic pathway, which represents a clathrin- and dynamin-independent internalization route [39]. ADAM17 and CD13 were recently described as intrinsic targets of RECK. In HT1080 epithelial cells, CD13 and membrane type 1-MMP are internalized with markers of both clathrin- and caveola-dependent endocytosis [43]. However, in the presence of RECK, both proteases are internalized preferentially *via* endocytosis that is neither clathrin- nor caveola-dependent [43]. Moreover, in gastric cancer cells, ADAM10 and ADAM17 are pulled down together by RECK - suggesting a physical interaction between RECK and ADAMs at the cell surface [46]. In the myeloid compartment, RECK is expressed by human monocytes and peritoneal macrophages [47, 48]. In view of these observations, one can legitimately hypothesize that RECK modulates the internalization of CD13 and ADAM17 in AML cells. However, RECK has been shown to inhibit post-transcriptional MMP-2/-9 expression and proMMP-9 secretion [45, 49]. Here, we found that MY7 treatment did not affect proMMP-9 release by AML blasts and U937 cells. This observation strongly suggests that ADAM17 and CD13 are not internalized *via* the endocytic route used by RECK. Nevertheless, the exact mechanisms of ADAM17 and CD13 endocytosis in AML cells requires further investigation.

Endocytic pathways require specific lipid-containing structures [39]. The plasma membrane contains lipid rafts, which are small lipid microdomains enriched in cholesterol and glycosphingolipids [50]. Lipid rafts also contain various receptors, membrane transporters and signal-transducing kinases with functions in protein transport, cell polarization and signal transduction [50]. In T cells, for example, the membrane-anchored protease CD26 (dipeptidyl peptidase IV) is present in lipid rafts; anti-CD26 mAb leads to CD26 internalization and increases its recruitment with CD45 (a tyrosine phosphatase) to rafts, resulting in enhanced tyrosine phosphorylation of signalling molecules [51]. In various cell types including peripheral blood mononuclear cells, ADAM17 localizes to lipid rafts [52-56]. CD13 is also associated with lipid rafts in monocytes [57, 58]. It is therefore legitimate to suggest that both CD13 and ADAM17 are present in membrane rafts in AML cells. We have previously demonstrated that the PI3K/AKT and caspase-dependent signalling pathways are involved in MY7-mediated U937 cell apoptosis [28]. It is not known whether (i) ADAM17 and CD13 cooperate in inducing apoptosis in AML cells or (ii) ADAM17 directly contributes to the activation of signalling transduction pathways induced by MY7 treatment. Our preliminary data however indicate that anti-CD13-mediated apoptosis is observed in ADAM17^null/low^ CD13^+^ AML cells in 7/9 cases suggesting that MY7-mediated downregulation of ADAM17 is not a critical enabling event for anti-CD13 mediated apoptosis in AML cells.

Growth factors and cytokines produced by inflammatory cells contribute to tumor growth and angiogenesis [59]. Adhesion molecules (including integrins, immunoglobulin-like CAMs, selectins and CD44) and chemokines modulate migration and invasion of normal and tumoral cells [60]. ADAM17 is involved in the cleavage of numerous surface molecules [59, 61], most of which are considered to be relevant in tumour-associated processes such as cell growth, migration and invasion (TNF-α, CD44, L1-CAM, L-selectin, ICAM1, CX3CL1, etc..) ADAM17's functions in AML cells have yet to be characterized. AML cells are characterized by increased proliferation and survival, medullary and extramedullary invasion [1, 2]. Inhibition of ADAM17 blocks leukocyte migration through inflamed endothelium [61]. Accordingly, we speculate that ADAM17 contributes to AML progression by affecting surface molecules involved in leukocyte proliferation, recruitment and migration. Therefore, targeting ADAM17 by anti-CD13 may block the AML tumoral process. Finally, CD16 (FcγRIII) expressed by natural killer (NK) cells and involved in Ab-dependent cell cytotoxicity, is cleaved by ADAM17 [62, 63]. A humanized bispecific Ab containing binding sites for NK CD16 and AML CD33 triggers NK cell activation, thereby inducing NK cytotoxicity against AML cells [64]. ADAM17 inhibition prevents CD16 shedding and enhances NK cell activation and specificity against AML cells [64]. In the latter study, the existence of ADAM17 in AML cells was ignored. It is therefore legitimate to also suggest a role for AML ADAM17 in CD16 shedding. Thus, CD13 ligation by downregulating ADAM17 could have therapeutic potential in treating AML.

In conclusion, we consider that our observations are likely to contribute to a better understanding of the expression and regulation of ADAM17 in AML. Although CD13 and ADAM17 expression levels vary from one AML subtype to another, this does not preclude the possibility that these two metalloproteases may cooperate under certain conditions. Given that CD13 and ADAM17 are expressed in various human cancers, it may be of great value to establish the biological significance of CD13-ADAM17 association in this context.

## METHODS

### Chemicals and reagents

Anti-CD13 (MY7, mIgG1), anti-CD13 (SJ1D1, mIgG1), phycoerythrin (PE)-conjugated anti-CD13 (SJ1D1, mIgG1), PE-cyanin 5 (PC5)-conjugated anti-CD13 (mIgG1, Immu103.44), PE-mIgG1 and fluorescein isothiocyanate (FITC)-anti-CD33 (mIgG1, D3HL60.251), FITC-mIgG1, PC5-mIgG1 and mIgG1 were obtained from Beckman-Coulter (Luminy, France). Anti-CD13 WM15 (mIgG1) was from BD-Pharmingen (San Jose, CA, USA). PE-anti-ADAM17 (mIgG1, 111633, targeting the ectodomain) and anti-ADAM17 (mIgG1, 136121, targeting the cytodomain) were obtained from R&D Systems Europe (Abingdon, UK). Anti-ADAM17 (rabbit Ig, 3976) was from Cell Signaling Technology Inc. (New England Biolabs, Hitchin, UK). Anti-actin (C4, mIgG1) was from ICN Biomedicals (Aurora, OH, USA). Secondary antibodies were horseradish peroxidase-conjugated antibodies from Dako Cytomation (Glostrup, Denmark). Ala-para-nitroanilide was obtained from Sigma (Saint Louis, MO, USA). Abz-LAQAVRSSSR-Dpa was obtained from Calbiochem (Darmstadt, Germany). CD13 small interfering RNA duplex was from Santa-Cruz (Tebu-Bio, SA, France). Negative control siRNA was from Ambion (Austin, TX, USA).

### Patient samples

Leukemic blood samples from 52 treatment-naive AML patients (age range 18-80) were obtained from the “Tumorothèque Hématologie” biological resource centre at Saint-Antoine Hospital (Paris, France) after the provision of written, informed consent (*European Organisation for Research and Treatment of Cancer* formulary study #06012). The diagnosis of AML was established in accordance with standard clinical criteria and the FAB Committee's cytological criteria (≥ 80% peripheral blood AML blasts CD33^+^ CD13^+^) (M0: undifferentiated blast; M1: undifferentiated myeloblast; M2: myeloblast; M4: myelomonocyte; M5: monoblast). The study was conducted and monitored in compliance with the Declaration of Helsinki 2002. Ethics approval was given by the independent ethics committees at Saint-Antoine Hospital (Paris, France) and the French National Institute of Cancer (“Tumorothèque Hématologie” Paris-Saint-Antoine Hospital COHO0203 INCA 2007). Peripheral blood mononuclear cells (PBMCs) were separated by Ficoll-Hypaque density gradient (1.077 g/ml) centrifugation. Cells (10^6^/ml) were cultured in RPMI 1640 medium supplemented with 10% heat-inactivated foetal calf serum (FCS) (Gibco; lipopolysaccharide levels < 0.1 ng/ml), 2 mM L-glutamine, 1 mM sodium pyruvate and 40 μg/ml gentamycin (Gibco) in a 5% CO2 humidified atmosphere at 37°C.

### Cell lines

The mycoplasma-free AML U937 (ATCC CRL-1593.2; French-American-British/FAB phenotype M5, monoblast), NB4 (M3, promyelocyte) [65], HL-60 (ATCC 240-CCL; M2, myeloblast) and THP-1 (ATCC TIB-202; M5, monoblast) cells were cultured in complete RPMI 1640 medium supplemented with 5% FCS in a 5% CO2 humidified atmosphere at 37°C [66]. For every experiment, cells were harvested in log-phase proliferation at passage 16 or less. Cells (2 x 10^5^/ml) were treated with IgG1 or anti-CD13 mAbs (10 μg/ml) for various periods of time. Cell proliferation was evaluated by counting the number of viable cells (with diameters ranging from 9 to 14 m) in a Coulter Multisizer (Beckman-Coulter, Villepinte, France).

### Flow cytometry

Intact cells were directly immunostained with specific mAbs as described in [67]. Stained cells (30,000) were analyzed using a flow cytometer (Beckman-Coulter). Values are quoted as the percentage of positive cells and the mean of fluorescence intensity (MFI) corresponds to antigen relative density per cell (obtained by subtracting the peak channel number of the negative control from the peak channel number of the corresponding experimental sample).

### CD13 siRNA transfection

** U937 cells (1x10^6^) in 100 μl of solution kit V were transfected with CD13 ds siRNA or negative control ncs siRNA (10-100 nM) using an Amaxa nucleofector, according to the manufacturer's protocol (program U13). Cells were then plated in 6-well plates for 24, 48 or 72 h. Cells were harvested, counted, analyzed for CD13 and ADAM17 transcript and protein expression, and CD13 enzymatic activity as described below.

### RNA isolation, cDNA synthesis and PCR

RNA extraction from treated cells and cDNA synthesis were conducted as described [67]. The cDNAs for human A, MMP-2, MMP-9, TACE and β2-microglobulin were amplified by PCR, and the primers were synthesised by Sigma-Proligo (Sigma-Proligo France) according to published sequences [68-72]. The PCR products were visualized by electrophoresis in 1.8% agarose gel containing 0.2 μg/ml ethidium bromide. The bands were acquired in an Appligen densitometer (Oncor).

### Immunoprecipitation experiments and Western blotting

For coimmunoprecipitation experiments as previously described [73], cells were lyzed in 1% CHAPS, 20 mM Tris-HCl pH 8.0, 150 mM NaCl and a cocktail of protease inhibitors. Cell lysates were pre-cleared for 30 min at 4°C with Protein G-agarose beads (Santa Cruz Biotechnology) equilibrated with lysis buffer in the presence of isotype antibody mIgG1. After incubation of pre-cleared lysates 6 h at 4°C with the relevant antibodies (anti-CD13 MY7 or anti-ADAM17/R&D 136121), protein A/G-agarose was added and gently rocked overnight at 4°C. Immunoprecipitates (IP) were washed with 150 mM NaCl, 10 mM Tris-HCl pH7.4, and mixed with the electrophoresis sample buffer containing 200 μM β-mercaptoethanol (3 min at 95°C). Total cell extracts and IP were separated on 7.5 % SDS-PAGE, transferred to nitrocellulose and blotted as described previously [73] Immunoblotting was performed with primary antibodies anti-CD13 (SJ1D1) or anti-ADAM17 (R&D 3976) and samples were then incubated with HRP-coupled secondary antibodies. Blots were visualized with an enhanced chemiluminescence kit (GE Healthcare Europe, Saclay, France).

### Measurement of MMP-2/-9 gelatinolytic activity by zymography

Analysis of proMMP-2 and proMMP-9 activities was carried out in 7.5% (w/v) SDS-polyacrylamide gels containing 0.1% gelatin (w/v) as described in [71]. Gelatinolytic activities of MMPs were detected as transparent bands on the background of Eza-blue stained gelatin. The bands were acquired in an Appligen densitometer (Oncor).

### ELISA analysis

The culture supernatants from primary AML cells or U937 cells and plasma from patients with AML were harvested under sterile conditions and frozen before MMP-2, MMP-9, ADAM17 and IFN-γ contents were determined using commercial ELISA kits provided by R&D (Abingdon, UK). Controls included FCS-supplemented RPMI 1640 medium alone incubated under the same conditions. Detection level for MMP-2 and MMP-9 was 1 ng/ml, for ADAM17 10 pg/ml and for IFN-γ 8 pg/ml.

### Statistics

Data are presented as means ± SD from n independent experiments. A two-tailed, paired Student's *t*-test was used to compare test and control groups. The threshold for statistical significance was set to *P* < 0.001.
